# Immune checkpoint inhibitor-induced anti-Hu antibody-associated gastrointestinal pseudo-obstruction: a case report and literature review

**DOI:** 10.3389/fimmu.2025.1555790

**Published:** 2025-04-01

**Authors:** Guang-Qing Shi, Heng-Ning Lian, Xue Wang, Jie-Qiang Xia, Huan Wang, Li-Jie Ma, Zhen-Liang Xiao, Jing Zhou

**Affiliations:** ^1^ Department of Respiratory and Critical Care Medicine, The General Hospital of Western Theater Command, Chengdu, Sichuan, China; ^2^ Department of Gastroenterology, The General Hospital of Western Theater Command, Chengdu, Sichuan, China; ^3^ Department of Neurology, The First People’s Hospital of Shuangliu District, Chengdu, Sichuan, China

**Keywords:** anti-Hu antibodies, gastrointestinal pseudo-obstruction, immune checkpoint inhibitors, serplulimab, paraneoplastic neurological syndrome

## Abstract

The immune checkpoint inhibitors (ICIs)-induced anti-Hu antibody-associated gastrointestinal pseudo-obstruction (GIPO) is a paraneoplastic neurological syndrome related to autoantibodies. It has a very low incidence but a high mortality rate. This report presents the case of a patient with extensive-stage small-cell lung cancer who developed recurrent bowel obstruction symptoms following ICI therapy. Colonoscopy and abdominal CT tomography failed to identify the underlying cause. A definitive diagnosis of GIPO was made based on the histological findings from an exploratory laparotomy and serum levels of paraneoplastic antibodies. Despite treatment with corticosteroids, no significant improvement was detected in the symptoms, and the patient ultimately died. This case highlights the challenges of managing this rare complication. When unexplained bowel obstruction occurs during ICI therapy, antineuronal antibody testing should be performed to exclude GIPO, as early identification and intervention can reduce mortality.

## Introduction

Lung cancer is the most frequently diagnosed cancer worldwide. In 2022, roughly 2.48 million new lung cancer cases were diagnosed globally, resulting in approximately 1.81 million fatalities (1). Since the approval of the first programmed death protein-1 (PD-1) inhibitor in 2014, ICIs have revolutionized lung cancer therapy, with combination regimens now achieving 5-year survival rates of 15–20% in advanced lung cancer (2). However, their expanded use has unmasked previously unrecognized immune-mediated toxicities, particularly in neurological domains. A systematic review showed that among 305,879 patients treated with ICIs, 58,291 experienced immune-related adverse events (irAEs), with an incidence rate of 19.1% (3). These adverse reactions affect almost all body systems, with immune-related neurological adverse events being relatively rare complications. The overall incidence rate of neurological irAEs is approximately 3.8% for anti-CTLA-4 therapy, 6.1% for anti-PD-1 therapy, and 12% for a combination of both therapies. The incidence rate of severe neurotoxicity was <1.0% (4), including paraneoplastic neurological syndromes (PNS), as due to its low incidence, clinical awareness is generally insufficient.

The PNS encompasses a collection of disorders linked to cancer that may affect different areas of the central or peripheral nervous system. These conditions do not result from the local effects of cancer or its spread within the nervous system; rather, they stem from immune responses triggered by cancer, primarily focusing on neuronal proteins (5). The clinical manifestations and antibody expression types in PNS are diverse. High-risk neurological phenotypes include encephalomyelitis, limbic encephalitis, rapidly progressive cerebellar syndrome, opsoclonus-myoclonus syndrome, subacute sensory neuronopathy, gastrointestinal pseudo-obstruction (GIPO), and Lambert–Eaton myasthenic syndrome (6), all of which have high mortality and morbidity rates (7). The immune responses involved encompass various antibodies, among which anti-Hu antibodies (anti-neuronal nuclear antibody type 1) represent a significant subtype of paraneoplastic anti-neuronal antibodies. These autoantibodies specifically target the Hu protein family within neuronal nuclei (8). The Hu protein family, comprising HuD, HuC, and Hel-N1, is primarily localized in neurons and neuroendocrine cells, where it participates in the regulation of mRNA stability and translation processes. The production of anti-Hu antibodies is typically associated with autoimmune reactions linked to tumors, particularly small-cell lung cancer (SCLC), wherein these antibodies attack the nervous system, leading to PNS (9). Previously, we reported a case of SCLC wherein the patient who tested positive for anti-Hu antibodies following adebrelimab treatment presented with peripheral neuropathy (10). In addition, anti-Hu antibody-associated PNS can manifest as cerebellar ataxia, limbic encephalitis, as well as GIPO. GIPO is an uncommon disorder that primarily affects the extrinsic nerves associated with the gastrointestinal tract, myenteric plexus, and pacemaker cells of Cajal (11). Clinically, it presents with recurrent abdominal pain, bloating, constipation, and/or vomiting, as well as abnormal gastric emptying or increased small intestinal pressure, but with no evidence of mechanical obstruction (12). Serplulimab is an Fc-engineered humanized IgG4κ monoclonal antibody that selectively targets the PD-1 receptor with high affinity. By effectively blocking the immunosuppressive interaction between PD-1 and its ligands PD-L1/PD-L2, this therapeutic agent restores T-cell-mediated antitumor activity (13). This report presents a case of serplulimab-induced anti-Hu antibody-associated GIPO to increase awareness of ICI-induced gastrointestinal-related PNS.

## Case report

**Figure 1:**
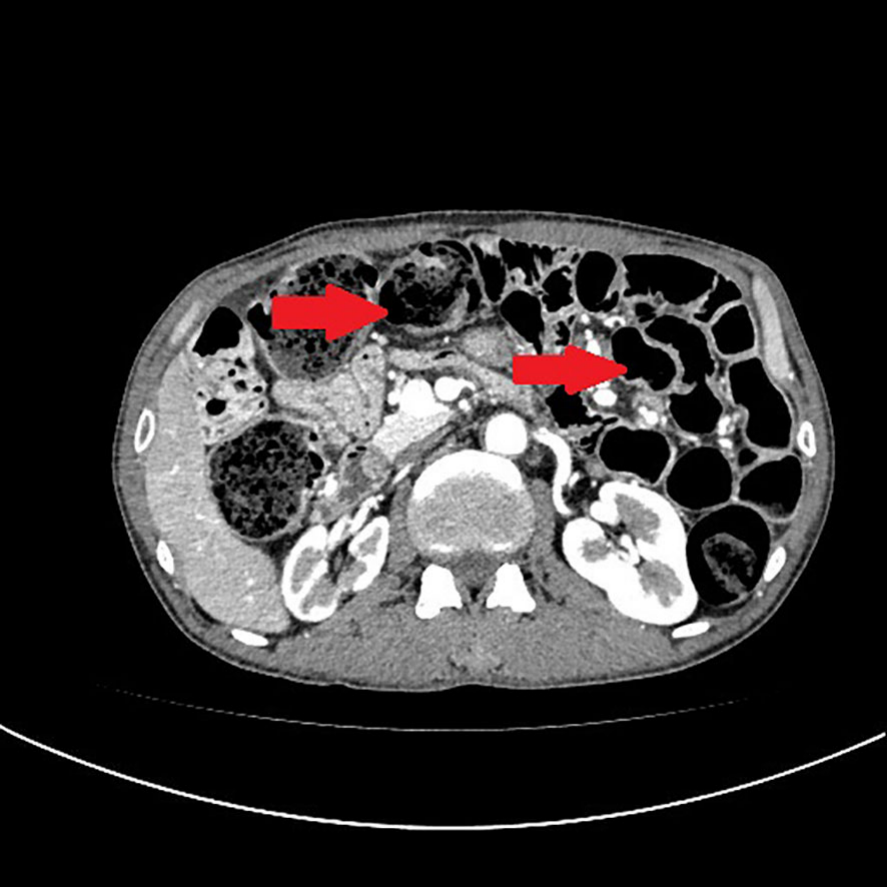
Abdominal computed tomography (CT) reveals diffuse dilatation and gas accumulation in the bowel, particularly the colon, suggesting incomplete intestinal obstruction.

**Figure 2:**
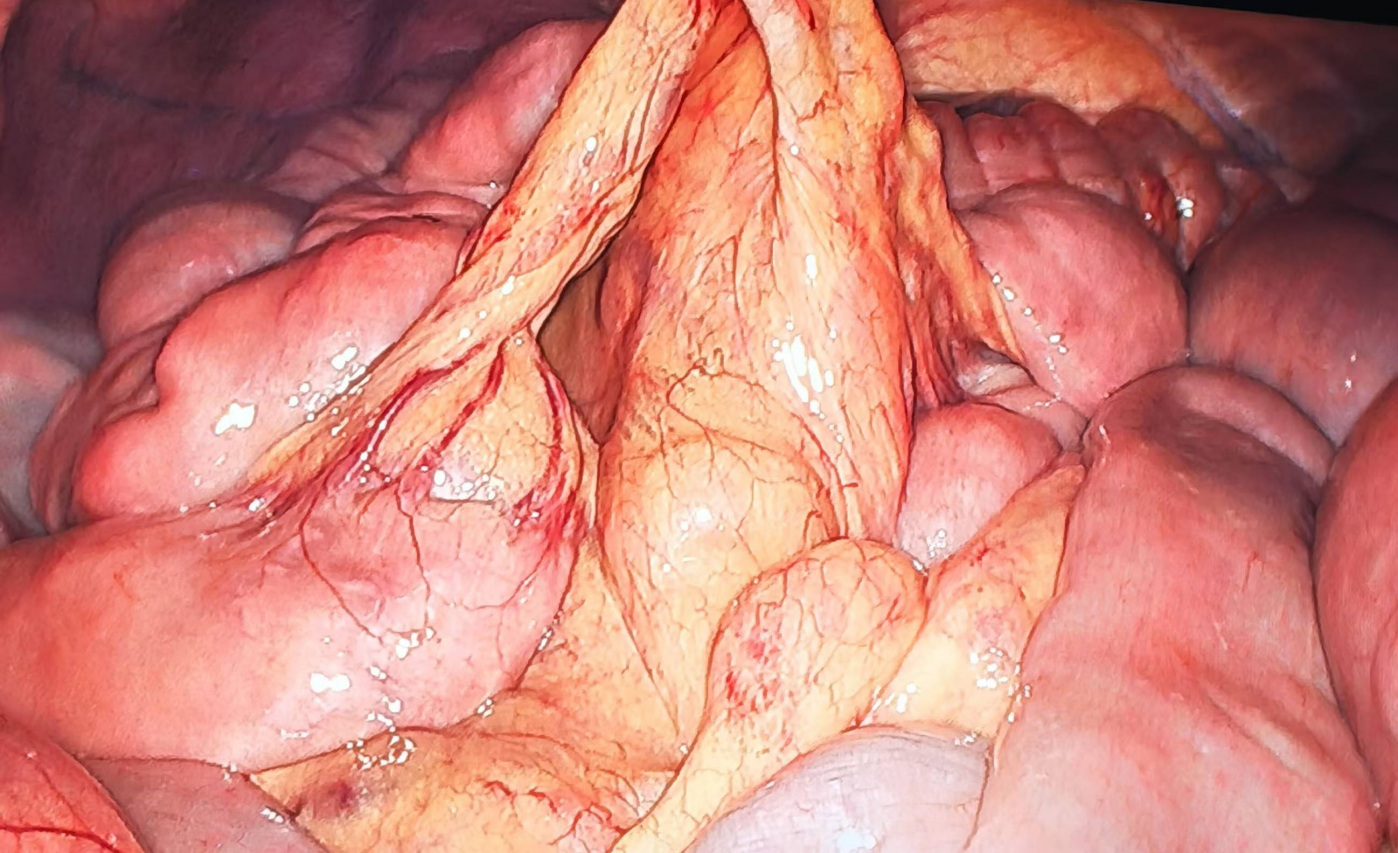
Intraoperative findings show significant bowel dilation and gas accumulation with no evidence of small bowel tumors or inflammatory diseases. The colon and rectum appear normal, with slight swelling of the small bowel mesentery and numerous enlarged lymph nodes.

**Figure 3:**
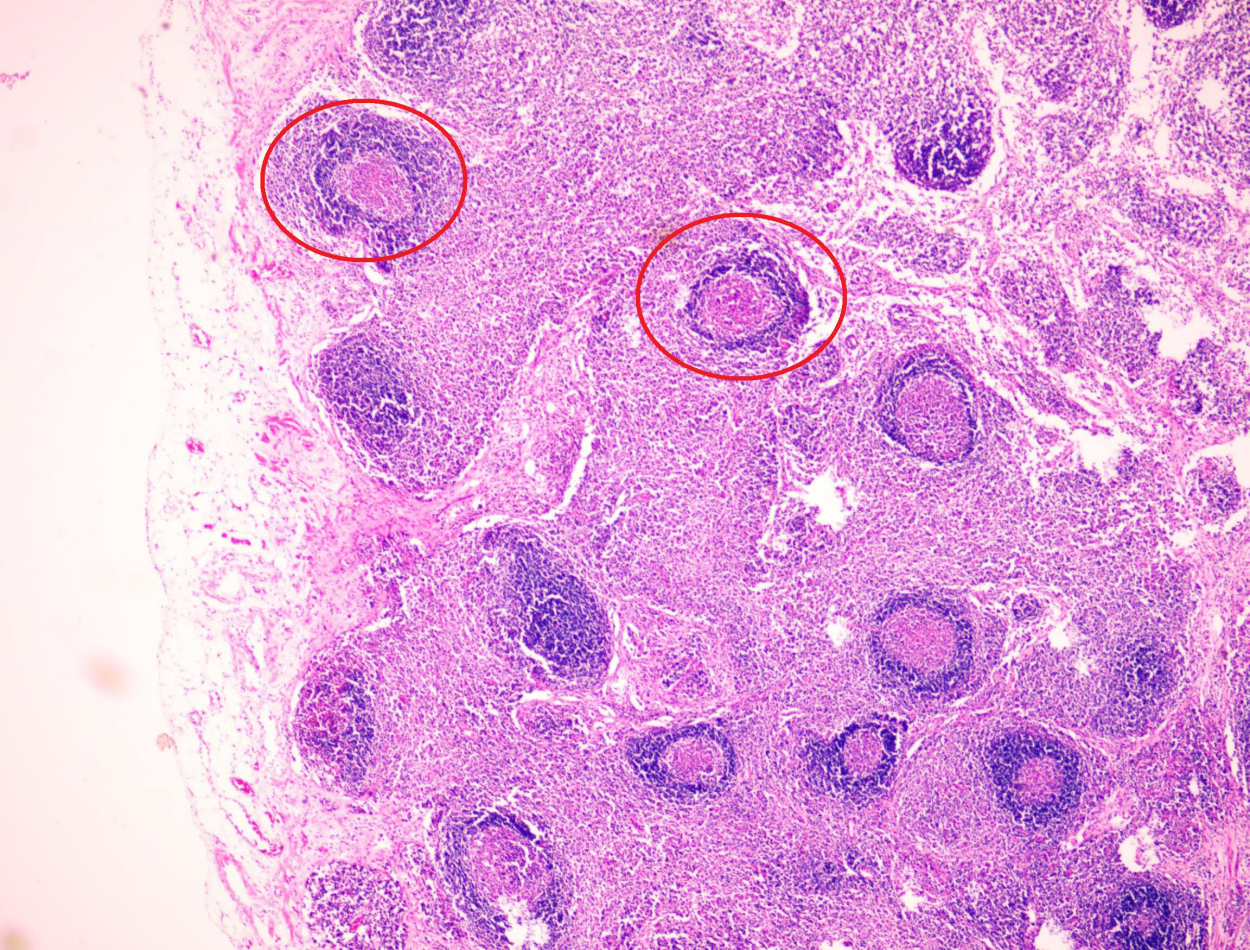
Pathological examination of the lymph nodes reveals reactive hyperplasia.

A 54-year-old male patient presented with facial and dorsal hand edema in July 2023. He was diagnosed with SCLC with hilar, mediastinal lymph node, and pleural metastases (cT1N3M1a, extensive stage) and a PS score of 1. Chemotherapy commenced on August 3, 2023, with four cycles of intravenous etoposide 160 mg (days 1–3), carboplatin (400 mg) on day 1, and serplulimab 300 mg on day 1 (Q3W). Subsequently, the patient received serplulimab maintenance therapy (Q3W). Follow-up evaluations after 18 cycles showed partial response (PR). However, after two cycles of serplulimab treatment, the patient intermittently (every 2–3 months) experienced hard stools and occasional constipation. These symptoms were not considered significant at the time and were relieved with glycerin suppositories. However, after completing 18 cycles (September 9, 2024), he was admitted to our gastroenterology department due to “no bowel movement for 5 d.” Abdominal computed tomography (CT) revealed diffuse dilatation and gas accumulation in the bowel, particularly the colon, suggesting incomplete intestinal obstruction (**Figure 1**). Gastroscopy showed chronic atrophic gastritis (C2) with bile reflux, and colonoscopy revealed a subpedunculated polyp approximately 1.5 cm in size, 20 cm from the anus, with smooth mucosa in the sigmoid colon. The scope reached the descending colon at 40 cm, where the mucosa and colonic haustra were smooth; however, due to severe pain and copious dry feces, the procedure was terminated. Despite multiple treatments with enemas and medications, the patient continued to experience an inability to defecate; in over 2 months, he lost 20 kg in weight. On November 12, 2024, the patient underwent laparoscopic exploration and mesenteric lymph node biopsy under general anesthesia. Intraoperative findings included significant bowel dilation and gas accumulation with no evidence of small bowel tumors or inflammatory diseases. The colon and rectum appeared normal, with slight swelling of the small bowel mesentery and numerous enlarged lymph nodes (**Figure 2**). Pathological examination of the lymph nodes revealed reactive hyperplasia (**Figure 3**), suggesting a functional gastrointestinal disorder. Therefore, ICI-related GIPO was suspected. Testing for antibodies related to serum paraneoplastic syndrome using the CAN and TBA methods, we detected anti-Hu antibodies at a dilution of 1:1000. However, tests for other antibodies against Ri, CV2, Amphiphysin, Ma1, Ma2, SOX1, DNER, Zic4, Titin, Recoverin, PKC, GAD65, and Yo were negative. The family chose not to proceed with lumbar puncture for cerebrospinal fluid antibody testing. Thus, the patient was diagnosed with anti-Hu antibody-related GIPO induced by serplulimab. Following this diagnosis, serplulimab was discontinued, and the patient received intravenous methylprednisolone sodium succinate at a dosage of 40 mg daily for 5 d. No symptom relief was observed, and the family subsequently declined high-dose steroid pulse therapy (1000 mg). The patient died 22 d later, and an autopsy was not performed at the family’s request.

## Discussion

Adverse events and toxicities related to ICIs are referred to as irAEs (14). Diarrhea and colitis are common gastrointestinal irAEs with an incidence rate of approximately 13.8%. They are easily identifiable and can be promptly managed in clinical practice (15). However, irAEs presenting as bowel obstruction are rare and can be misdiagnosed as symptoms of tumor metastasis or cachexia-induced digestive issues (16). In this case, GIPO was diagnosed in the absence of evidence of mechanical or vascular obstruction. GIPO diagnosis in patients with cancer requires excluding chemotherapy-related ileus, radiation-induced intestinal paralysis, infectious enteritis, peritoneal metastasis, and carcinomatous peritonitis, then paraneoplastic syndrome antibody testing must be performed clarify the diagnosis further (17). GIPO results from neuromuscular dysfunction of the gastrointestinal tract, which can be caused by neural inhibition, toxin stimulation, or smooth muscle pathology in the intestinal wall, leading to motility disorders. In cancer, GIPO is primarily associated with PNS in patients with lung cancer, neuroblastoma, and neuroendocrine tumors and is often linked to anti-Hu positivity. Most previous reports on GIPO in ICI-treated patients with cancer do not include antibody testing due to insufficient disease comprehension (18–22). Only four studies reported anti-Hu antibody testing, two each with positive and negative results (**Table 1**) (23–26). This suggests that when ICI-treated patients with cancer develop neurological symptoms, autoantibody testing and a comprehensive PNS assessment should be performed to confirm the diagnosis. According to the PNS diagnostic criteria proposed by Graus et al. (6), a scoring system (PNS-Care Score) has been designed based on the patient’s clinical phenotype, presence of neuronal antibodies, and association with cancer. A score of ≥8, along with excluding other possible diseases, confirms PNS diagnosis.

**Table 1:** Literature review of immune checkpoint inhibitor-induced gastrointestinal pseudo-obstruction.

Age/Sex	Tumor	ICI (number of cycles)	Treatment	Antibody	Outcome	Reference
66/Male	MCC	Ipilimumab Nivolumab (1)	Methylprednisolone (2 mg/kg) Tacrolimus Plasmapheresis	Negative	Death (autopsy)	Appelbaum et al. (23)
66/Male	SCLC	Sintilimab (2)	Methylprednisolone (1 mg/kg)	Anti-Hu antibody (CSF)	Improved	Kang et al. (24)
75/Male	SCLC	Durvalumab (1)	Methylprednisolone (1,000 mg)	Anti-Hu antibody (serum)	Death	Saitou et al. (25)
71/Female	GOJ	Pembrolizumab (8)	Methylprednisolone (unknown)	Negative	Improved	Besaw et al. (26)
79/Female	MM	Nivolumab (9) Ipilimumab (1)	Methylprednisolone (1 mg/kg)	Unknown	Improved	Ishibashi et al. (18)
62/Male	NSCLC	Nivolumab (14)	Methylprednisolone (2mg/kg)	Unknown	Improved	Fragulidis et al. (19)
56/Male	NSCLC	Nivolumab (6)	Methylprednisolone (2 mg/kg) infliximab	Unknown	Improved	Dai et al. (20)
67/Male	SCLC	Atezolizumab (1)	Methylprednisolone (1.5mg/kg)	Unknown	Improved	Trontzas et al. (21)
64/Male	HCC	Pembrolizumab (6)	Methylprednisolone (40mg)	Unknown	Improved	Qian et al. (22)
54/Male	SCLC	SerpluLimab (18)	Methylprednisolone (40mg)	Anti-Hu antibody (serum)	Death	Present case

ICI, Immune checkpoint inhibitors; MCC, Merkel cell carcinoma; GOJ, Gastro-esophageal junction; MM, Metastatic melanoma; NSCLC, Non-small-cell carcinoma; SCLC, Small-cell lung carcinoma; HCC, Hepatocellular carcinoma.

The challenge in GIPO diagnosis is further highlighted in epidemiologic data. An Italian epidemiological study conducted between 2009 and 2017 showed that none of the 89 patients with PNS presented with GIPO (27). Similarly, among 264 patients with SCLC in the UK, 24 developed PNS between 2005 and 2010; however, none had GIPO (28). To the best of our knowledge, only two reported cases of ICI-induced anti-Hu-associated GIPO have been documented (24, 25). In the present study, three instances were reported, of which, all developed during SCLC treatment, which may be related to the higher proportion of patients with SCLC-developing PNS (29). In known cases of Hu-Ab-associated PNS, the condition typically progresses rapidly over weeks to months, leading to a poor prognosis, with more than half of the patients becoming bedridden or requiring a wheelchair and only 5–7% showing improvement, with a median survival time is less than a year (30, 31). Reports have suggested that age, cancer-related frailty due to advanced disease, and severe neurological manifestations are associated with Hu-Ab syndrome-related mortality following ICI treatment (30). Unlike other neurological irAEs, Hu-Ab-positive patients often show no symptom relief even after discontinuing the ICI and receiving immunosuppressive treatment, suggesting a unique immunopathological mechanism that urgently requires elucidation (32). The clinical features and treatment course, in this case, provide key clues for understanding ICI-related GIPO. Gastrointestinal endoscopy and abdominal exploration were performed to eliminate other causes. The patient had a high anti-Hu antibody titer and PNS-Care score of 10 points (≥8 points can be diagnosed). In contrast to previous cases, our patient presented with only mild constipation at the beginning, which was relieved briefly after symptomatic treatment, delaying GIPO diagnosis.

Comparison of treatment strategies further revealed key differences in clinical decision-making. Primary therapies for ICI-induced PNS include corticosteroids, intravenous immunoglobulins (IVIG), and plasmapheresis. Second-line treatment options include rituximab, cyclophosphamide, and mycophenolate (33). Plasmapheresis has also been used to accelerate ICI clearance, but the appropriate timing for this therapy remain unclear (34). An autopsy report by Appelbaum et al. reported a severe reduction in the number of sympathetic ganglia throughout the gastrointestinal tract, with no sympathetic ganglia present in the myenteric plexus and only a few remaining in the submucosa, suggesting that treatment may be ineffective after the disease has reached the “burned-out” stage (23). Therefore, treatment should be initiated immediately upon the onset of symptoms. In another previous report by Kang et al., three patients received methylprednisolone treatment, but only one survived (24). This patient had developed encephalitis and GIPO sequentially following the use of sintilimab. Since the patient had been treated with methylprednisolone for encephalitis before being diagnosed with GIPO, it was speculated that the early therapeutic intervention with corticosteroids may have contributed to the improvement in GIPO (24). While corticosteroids effectively mitigate inflammation, they cannot repair necrotic tissue or regenerate neurons, which might explain poor response to cortisol therapy. Furthermore, aggressive immunosuppressive therapy has demonstrated limited utility in severely disabled patients with PNS and antineuronal antibodies (35). Delayed administration may also permit inflammatory cascades, such as cytokine storms and oxidative stress, inflicting irreversible damage. Additionally, corticosteroids predominantly suppress innate immunity and humoral responses, rendering them insufficient for targeting T-cell-driven pathologies. In this case, the nerve damage was irreversible due to delayed treatment and suboptimal corticosteroid dose.

IVIG administered at 2 g/kg divided over 2–5 d can alleviate steroid-refractory GIPO. The primary mechanisms involve the neutralization of autoantibodies and immunomodulatory effects, thereby ameliorating impaired neuronal transmission (36). Jacob et al. (23) reported the successful management of a case of GIPO induced by ipilimumab plus nivolumab combination therapy through plasmapheresis, a strategy aimed at clearing circulating autoantibodies and pro-inflammatory mediators. Second-line treatment options for refractory autoimmune GIPO include rituximab and vedolizumab (37), while the use of cyclophosphamide and mycophenolate mofetil has not yet been reported. In the cases shown in **Table 1**, all patients were treated with methylprednisolone at a single dose of approximately 1–2 mg/kg. Only one patient received pulse steroid therapy (1000 mg/d). One case was managed with a combination of tacrolimus and plasmapheresis; however, the patient did not survive. In this case, due to a long-standing digestive system disease, the patient developed significant cachexia. The family refused high-dose corticosteroid pulse therapy and second-line biological treatment, and the patient eventually died.

Previous studies have indicated that the prognosis of ICI-induced GIPO is closely associated with the antibody profiles and dynamic changes in titers, although the findings are inconsistent. Vogrig et al. demonstrated that elevated titers of anti-Hu/Yo antibodies (>1:320) positively correlate with severe intestinal dysmotility, glucocorticoid resistance, and an increased risk of disease recurrence (9).In serum AQP4 antibody-positive patients with NMOSD, the neuronal autoantibody titers decreased, sometimes even reaching negative values, following high-dose glucocorticoid pulse and immunosuppressive therapies during the acute phase, especially in patients with low pre-treatment titers (<32). Similarly, in patients with high pre-treatment titers (>320), immunoadsorption treatment reduced the autoantibody titers gradually (38, 39). During maintenance immunotherapy, antibody titers gradually decline and may even turn negative, but some patients who have unchanged titers have a higher relapse rate compared to those who do (40). Lower titers of classic paraneoplastic syndrome-related antibodies may indicate a lower risk of associated tumors and better prognosis (41). In refractory patients with NMDAR encephalitis, no significant effect is depicted when first-line therapy is administered during the acute phase, whereas early use of second-line therapy is significantly important for reducing antibody titers (42, 43). Notably, in the present case, the high anti-Hu antibody titer (1:1000) was associated with an unfavorable clinical outcome.

This case report provides critical insights into the clinical management of ICI-induced GIPO, further underscoring the necessity for early recognition and risk stratification of irAEs in cancer immunotherapy. Research indicates that specific ICI regimens and baseline patient characteristics may significantly influence GIPO risk. Cuzzubbo et al. demonstrated through large-scale clinical data analysis that, compared to PD-1/PD-L1 monotherapy, CTLA-4 inhibitors (e.g., ipilimumab) or CTLA-4/PD-1 combination therapy substantially increase the incidence of severe irAEs (including GIPO) (4). Patients with pre-existing autoimmune neuropathies (e.g., myasthenia gravis) or gastrointestinal motility disorders appear more susceptible to such complications, though this remains speculative, with no confirmatory studies to date.

Effective prevention systems should be established for these high-risk populations in clinical practice via (1) baseline risk assessment with systematic screening for autoimmune history and anti-neuronal antibody testing in suspected cases before treatment initiation. (2) Personalized dose adjustment by considering extended dosing intervals or reduced doses for high-risk patients, as CTLA-4 inhibitors exhibit dose-dependent irAE profiles. For instance, ipilimumab dose escalation from 3 mg/kg to 10 mg/kg increases irAE incidence from 29% to 46% (44). (3) Dynamic monitoring and tiered intervention: Clinicians should remain vigilant for early symptoms such as abdominal distension, refractory constipation, and vomiting, implementing severity-based management. Mild cases warrant temporary ICI suspension with oral corticosteroids, whereas moderate-to-severe cases require hospitalization for intravenous steroid pulse therapy. Second-line interventions (e.g., IVIG or plasmapheresis) should be initiated if no response occurs within 72 hours. Multidisciplinary team collaboration can optimize decisions regarding ICI interruption/permanent discontinuation by comprehensively evaluating intestinal motility and immune injury mechanisms, thereby balancing antitumor efficacy and safety. Notably, GIPO profoundly impacts both patients’ quality of life and mental health. The HRQoL scores in patients with GIPO were significantly lower than those in general cancer populations, correlating strongly with symptom frequency and often triggering anxiety/depression (45).

## Conclusion

This case is a unique presentation of GIPO associated with anti-Hu antibodies in patients with EC-SCLC treated with the PD-1 inhibitor serplulimab. Early diagnosis of enteric neuropathy related to PNS is challenging and can be easily confused with tumor-induced gastrointestinal adverse events. Therefore, increasing the awareness of anti-Hu antibody-associated GIPO is crucial for identifying this rare condition and initiating early intervention. If the condition is severe, it is essential to promptly discontinue ICIs and consider the use of corticosteroids or other immunosuppressants based on the clinical situation to improve patient outcomes.

## Data Availability

The raw data supporting the conclusions of this article will be made available by the authors, without undue reservation.
